# Tumor necrosis-informed prognostic nomogram for clear cell renal cell carcinoma model development and clinical validation

**DOI:** 10.1186/s12894-025-01940-2

**Published:** 2025-10-10

**Authors:** Zhiwei Guo, Huiyu Zhou, Dingyang Lv, Zhao Hou, Jinshuai Li, Mohan Jia, Hongyang Du, Yingbo Kang, Qiwei Wang, Yabin Wang, Luyue Kou, Hanguang Fang, Zhengkun Wang, Weibing Shuang

**Affiliations:** 1 Department of Urology, First Hospital of Shanxi Medical University, Taiyuan, Shanxi Province China; 2 First Clinical Medical College of Shanxi Medical University, Taiyuan, Shanxi Province China; 3 Academy of Medical Sciences, Shanxi Medical University, Taiyuan, Shanxi Province China

**Keywords:** Tumor necrosis, Nomogram, Prognostic, Clear cell renal carcinoma

## Abstract

**Background:**

This study investigated the prognostic role of tumor necrosis (TN) in non-metastatic clear cell renal cell carcinoma (ccRCC).

**Methods:**

We enrolled 1,212 non-metastatic ccRCC patients undergoing nephrectomy (2013–2023) in this retrospective study. Computer-generated randomization allocated cases to derivation (70%, *n* = 848) and validation cohorts (30%, *n* = 364). Kaplan-Meier methodology compared overall survival (OS), cancer-specific survival (CSS), and recurrence-free survival (RFS) across TN-positive and TN-negative cohorts, with intergroup differences evaluated by log-rank testing. Prognostic determinants were identified through univariate and multivariate Cox proportional hazards regression. We developed a prognostic nomogram through stepwise Cox regression that integrated TN status with key clinicopathological variables. Validation employed: Harrell’s C-index, time-dependent ROC curves, calibration plots, and decision curve analysis (DCA).

**Results:**

TN positivity was significantly associated with reduced OS (HR: 2.12, 95% CI: 1.65–2.73; *P* < 0.001), CSS (HR: 2.45, 95% CI: 1.82–3.29; *P* < 0.001), and RFS (HR: 1.89, 95% CI: 1.32–2.70; *P* = 0.003) in multivariate analysis. The prognostic nomogram demonstrated excellent discrimination in the validation cohort, with C-indices of 0.855 (OS), 0.870 (CSS), and 0.724 (RFS). Time-dependent ROC analysis revealed robust predictive accuracy for OS at 1- (AUC: 0.892), 3- (AUC: 0.846), and 5-year (AUC: 0.826) intervals. Calibration curves demonstrated excellent consistency between predicted probabilities and actual outcomes. Decision curve analysis further revealed greater clinical net benefit than pT stage system and WHO/ISUP classification.

**Conclusion:**

TN is an independent prognostic marker in non-metastatic ccRCC. The novel nomogram integrating TN provides reliable risk stratification, aiding personalized postoperative management.

## Introduction

Renal cell carcinoma(RCC) constitutes 2–3% of all adult cancers and represents the third most frequent urinary malignancy, demonstrating steadily rising incidence rates throughout the past thirty years [1, 2]. RCC contributes to an estimated 403,000 new cases and 175,000 deaths globally each year [3]. Enhanced public health consciousness and sophisticated diagnostic technologies have progressively increased the proportion of incidentally detected renal cell carcinomas [4]. In RCC, ccRCC is the most common pathological type and has the worst prognosis compared with papillary renal cell tumor and chromophobe tumor. It has received increasing attention in clinical practice [5]. Although most cases of RCC are diagnosed at an early stage, approximately 20% of patients who undergo curative nephrectomy develop metastases during follow-up [6], and surgery is the primary treatment for localized RCC.Currently, although targeted therapy and immunotherapy extend the survival time of patients with advanced renal cell carcinoma, their efficacy is limited by their low objective response rate and drug resistance [7]. Consequently, evolving systemic therapies necessitate enhanced prognostic risk assessment for RCC patient stratification, clinical decisions, and survival optimization. Current prognostic cornerstones remain TNM staging and Fuhrman nuclear grading [8, 9]. In addition, prognostic models such as the American Joint Committee on Cancer (AJCC) staging system and the International Association of Uropathologists (ISUP) are also important factors influencing prognosis [10, 11]. While TNM staging and Fuhrman nuclear grading currently serve as core tools for prognostic assessment in ccRCC, they still have limitations in clinical practice. On one hand, these indices primarily rely on morphological features such as tumor size and invasion extent, making it difficult to capture the heterogeneity of tumor biological behavior—for instance, some patients with early-stage disease may still experience rapid progression [12]. On the other hand, for patients with similar pathological characteristics, traditional indicators fail to effectively distinguish prognostic risks, resulting in a lack of precise basis for clinical treatment decisions [13]. Therefore, there is an urgent need to integrate more biologically meaningful biomarkers to optimize prognostic stratification in ccRCC.TN is microscopic necrosis characterized by dead and degenerated cells, ghost cells, and apoptotic debris. It is defined as exceeding the cell’s own blood supply, leading to severe and chronic hypoxia and ultimately cell death [14–16].

As a hallmark of aggressive malignancies, tumor hypoxia manifests morphologically through necrosis [17]. This histological feature independently predicts adverse outcomes across multiple carcinomas, evidenced in breast [18], colorectal [19], and lung cancers [20].Evidence connects necrosis to three vascular aberrations: activated angiogenesis, deficient vascular maturation, and invasive vasculature [21], suggesting mechanistic involvement in metastatic dissemination. Within breast tumors, necrotic regions predict high-grade disease, larger diameter, ER-negative status, dense microvasculature, and macrophage accumulation [22–24].In the study of renal carcinoma, Khor et al. [15] and Ito et al. [25] reported that TN is closely related to the low survival rate of patients with renal carcinoma and should be used as an independent prognostic factor for patients with RCC.However, some studies have indicated that the prognosis of renal cell carcinoma is not associated with TN. Instead, TN serves as a negative predictor for the prognosis of renal cancer patients [26, 27]. Currently, the relationship between TN and the prognosis of renal cell carcinoma patients remains a subject of debate [25]. Existing studies have mostly focused on the independent prognostic value of TN, while research that integrates TN with clinicopathological features to develop visualized prognostic models remains scarce—hindering its direct application in clinical risk assessment [28]. As a result, the association between TN and patient prognosis has attracted significant attention within the medical community.

The aim of this retrospective study was to assess the prognostic significance of TN in non - metastatic ccRCC patients who underwent nephrectomy. In this research, we aim to assess the prognostic value of TN in ccRCC following renal carcinoma resection and determine whether TN could be utilized as a parameter for ccRCC invasiveness. Moreover, we further aimed to establish a clinicopathological model incorporating risk factors to predict the prognosis of ccRCC patients.

## Materials and methods

### Study population and research design

**Fig. 1:**
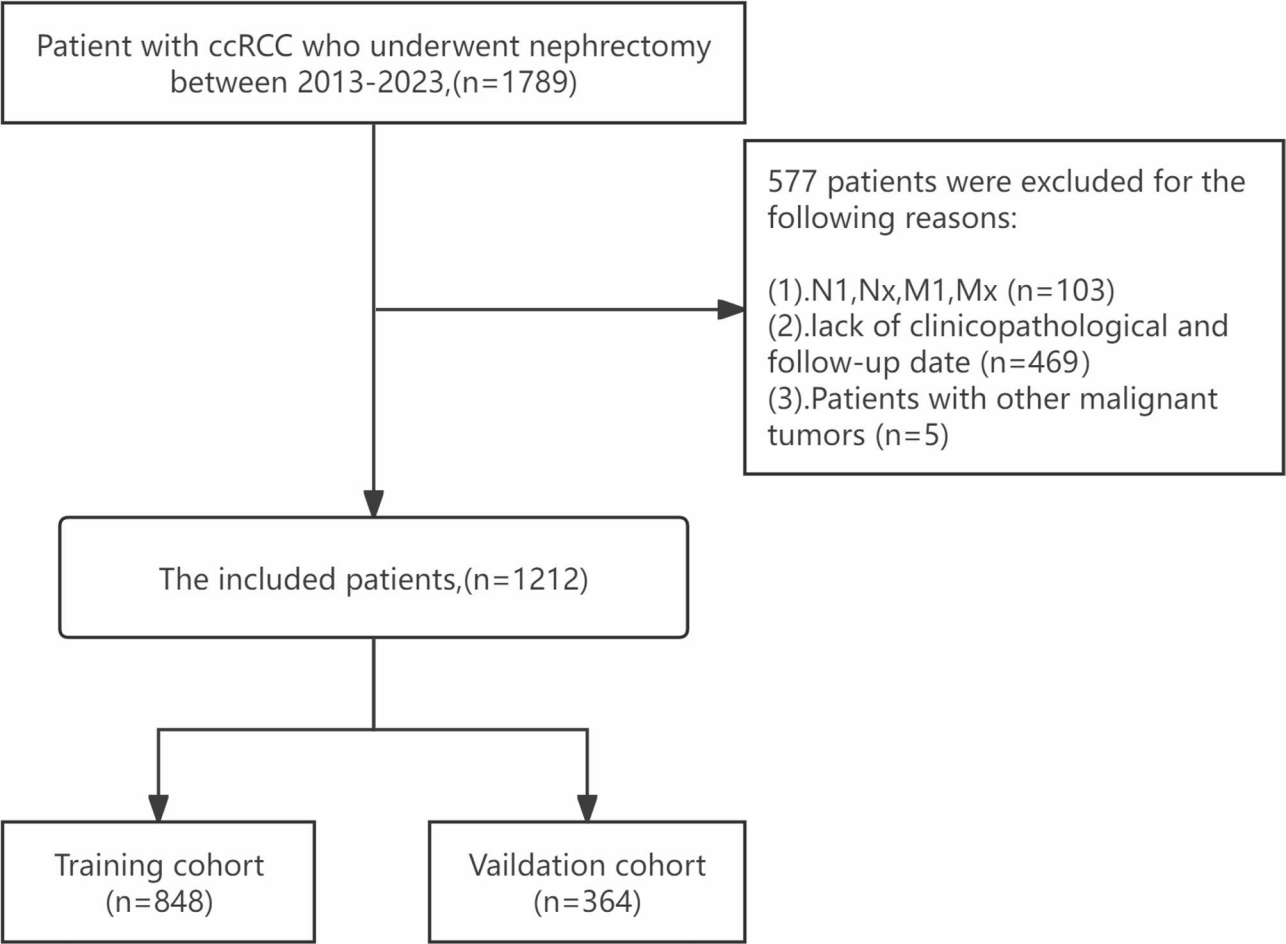
The flowchart of the ccRCC patients with training and validation cohorts

The study encompassed 1789 patients who underwent nephrectomy at the First Hospital of Shanxi Medical University from January 2013 to December 2023. The inclusion criteria were as follows: ① aged ≥ 18 years; ② patients who received radical nephrectomy or partial nephrectomy; ③ postoperative pathological confirmation of clear cell renal cell carcinoma; ④ no distant metastasis. The exclusion criteria were: ① lack of clinicopathological and follow - up data; ② co - existence of other malignancies; ③ receipt of radiotherapy, chemotherapy, or other anti - tumor immunotherapies. This study complied with the Declaration of Helsinki. The study was approved by the Ethics Committee of the First Hospital of Shanxi Medical University (Ethical Code: [2021] K048). The Institutional Review Board of the First Hospital of Shanxi Medical University waived the requirement for written informed consent. Ultimately, 1212 patients were included in this study. The specific details of the inclusion process are presented in Fig. 1.

### Data extraction

Clinicopathological data were collected through the electronic medical record system of our hospital. The dataset encompassed comprehensive demographic characteristics, clinical profiles, and histopathological parameters including: gender, age, anthropometric measurements (height, weight, and body mass index), smoking status, medical history (hypertension, diabetes, cardiovascular diseases, and cerebral infarction), tumor characteristics (pT stage according to the American Joint Committee on Cancer (AJCC) TNM staging system [29]; WHO/ISUP nuclear grading based on the 2016 WHO classification of renal cell tumors [30]; maximum tumor diameter), histopathological findings (presence of tumor necrosis, cystic change), and invasion patterns (renal sinus fat invasion, perirenal fat invasion, venous tumor thrombus, and neurovascular invasion).

### Follow - up

Patients underwent quarterly follow-ups for the initial 36 postoperative months, transitioning to semiannual evaluations during years 4–5, with annual surveillance thereafter.Assessments combined scheduled outpatient reviews and structured telephone interviews.Standardized evaluations included: Clinical history documentation, Physical examination, Laboratory analyses, Diagnostic imaging.Clinical outcomes included OS, CSS, and RFS. These outcomes were defined according to standard criteria in oncology research: OS was defined as the time from surgery to all-cause mortality or last contact; CSS referred to the interval from operation to RCC-attributed death (verified by attending physician and death certificate) or final follow-up; RFS denoted the duration from resection to radiologically/pathologically confirmed recurrence/metastasis or study termination [31].

### Statistical analysis

All patients (*n* = 1212) from 2013 to 2023 were randomly divided into a training cohort (*n* = 848) and a validation cohort (*n* = 346) at a ratio of 7:3. Independent - samples t - test and chi - square test were used to compare the clinicopathological variables between the training cohort and the validation cohort. The Cox proportional-hazards model was applied for univariate and multivariate analyses to identify independent prognostic factors [32]. Hazard ratios (HRs) and 95% confidence intervals (CIs) were calculated. Using the selected factors, nomogram models were established to estimate survival rates, with discriminative capacity quantified via area under the curve (AUC) and Harrell’s C-index [33]. Predictive accuracy was verified using bootstrap-corrected calibration curves (500 resamples). Clinical utility was evaluated through decision curve analysis (DCA) as described by Vickers et al. [34].

Survival distributions stratified by tumor necrosis status were compared using Kaplan-Meier methodology with log-rank testing.All analyses were performed in R 4.3.1 with specialized packages: Nomogram development/validation: rms.Regression modeling: survival.Time-dependent ROC: survivalROC.Clinical utility metrics: dcurves.Statistical significance threshold was defined as two-sided *P* < 0.05.

## Results

### Patient demographics

According to the inclusion and exclusion criteria, a total of 1212 ccRCC patients were included in this study. The patients from 2013 to 2023 (*n* = 1212) were utilized for the establishment and internal validation of the prediction model. Among these patients, 785 were male, accounting for 64.8%, and 427 were female, accounting for 35.2%. There were 553 elderly patients (aged ≥ 60 years), making up 45.6%, and 659 young patients (aged < 60 years), constituting 54.4%. Post - operative pathology revealed tumor necrosis in 175 cases (14.4%) and non - tumor necrosis in 1037 cases (85.6%). In terms of WHO/ISUP grade, 149 cases were grade I (12.3%), 751 cases were grade II (62.0%), 270 cases were grade III (22.3%), and 42 cases were grade IV (3.5%). Regarding tumor stage, 1004 patients were at T1 stage (82.8%), 163 at T2 stage (13.4%), 43 at T3 stage (3.5%), and 2 at T4 stage (0.2%). In terms of BMI, 559 cases had a normal BMI (46.1%), 628 cases were overweight (51.8%), and 25 cases were underweight (2.1%). Post - operative pathology reported intratumoral hemorrhage in 955 cases (78.8%), cystic change in 107 cases (8.8%), renal sinus fat invasion in 23 cases (1.9%), perirenal fat invasion in 9 cases (0.7%), and neurovascular Invasion in 14 cases (1.1%)0.686 cases (56.6%) had tumors smaller than 4 cm, 392 cases (32.3%) had tumors ranging from 4 to 7 cm, and 134 cases (11.1%) had tumors larger than 7 cm. 24 patients (1.9%) reported a feeling of a mass in the back. One hundred and 24 patients (10.2%) reported lower back pain, 53 patients (4.4%) had hematuria, 88 patients (7.3%) had cardiovascular diseases, 49 patients (4.9%) had a history of cerebral infarction, 334 patients (27.6%) had a smoking history, 291 cases (24.0%) had alcoholism, and 534 cases (44.1%) had a history of hypertension. Eventually, 82 patients (6.8%) died, and 95 patients (7.8%) experienced recurrence or progression (Table 1).

**Table 1:**
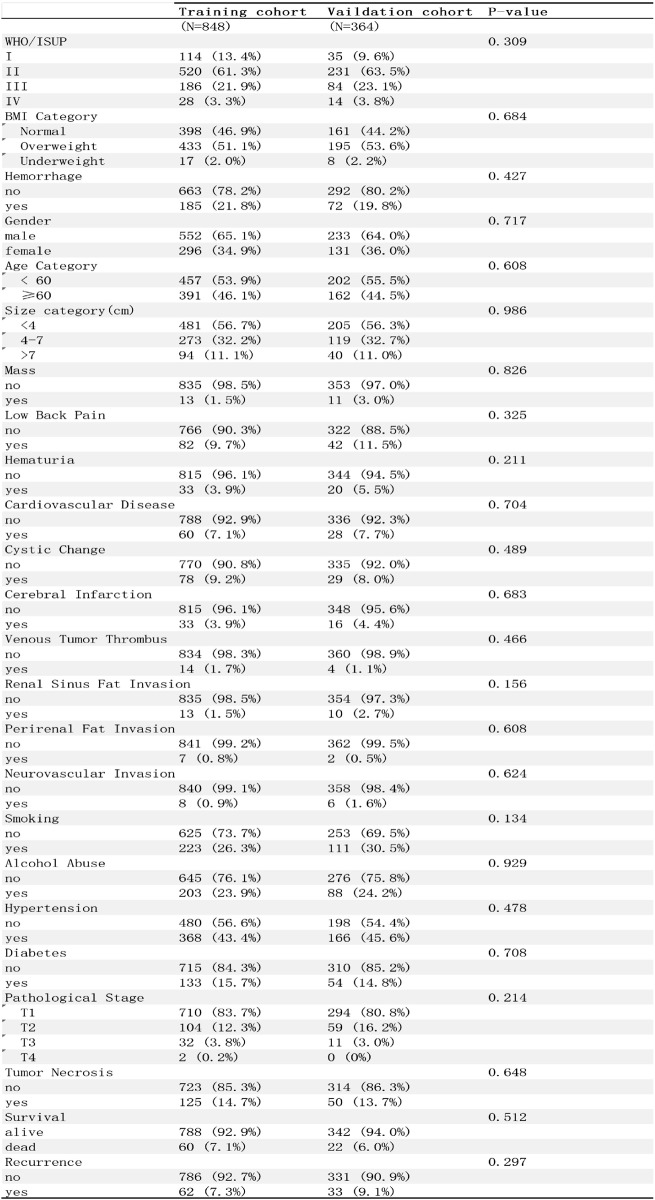
Clinicopathological characteristics of patients with ccRCC

### Univariate and multivariate Cox analyses

In the training cohort, univariate Cox regression analysis identified 12 significant risk factors, including TN, alcohol abuse, smoking, renal sinus fat invasion, venous tumor thrombus, WHO/ISUP grade, perirenal fat invasion, elderly patients (aged ≥ 60 years), diabetes, gender, T stage, and tumor size. Subsequently, we selected these factors to establish a multivariate Cox model to determine the independent prognostic factors. The results showed that diabetes(HR = 2.697, 95% CI:1.400-5.195, *P* = 0.003), alcohol abuse(HR = 2.660, 95% CI:1.417–4.99., *P* = 0.002), T stage(HR = 20.946, 95% CI:2.587-169.551, *P* = 0.004), tumor size(HR = 6.497, 95% CI:2.282–18.489, *P* < 0.001), TN(HR = 3.183, 95% CI:1.656–6.113, *P* < 0.001), elderly patients(HR = 2.740, 95% CI:1.470–5.103, *P* = 0.001), and WHO/ISUP grade(HR = 4.004, 95% CI:1.070-14.968, *P* = 0.039) were independent prognostic factors affecting the OS of patients(Table 2). Then, we used stepwise Cox regression and found that when the smoking variable was added to the model, the Akaike information criterion (AIC) value was the lowest (615.83), and the *p* - value of univariate Cox regression for smoking was less than 0.05. Therefore, we used the model with the added smoking variable. Meanwhile, univariate and multivariate analyses showed that TN(HR = 1.869, 95% CI:1.038–3.364, *P* = 0.037), gender(HR = 0.485, 95% CI:0.245–0.958, *P* = 0.037), T stage(HR = 16.720, 95% CI:2.71-103.138, *P* = 0.002), WHO/ISUP stage(HR = 4.407, 95% CI:1.300-12.598, *P* = 0.016), and tumor size(HR = 7.107, 95% CI:2.566–19.678, *P* < 0.001) were independent prognostic factors for the RFS of patients (Table 3). Diabetes(HR = 2.753, 95% CI:1.423–5.323, *P* = 0.003), alcohol-abuse(HR = 2.680, 95% CI:1.428–5.029, *P* = 0.002), T stage(HR = 20.022, 95% CI:2.463-162.752, *P* = 0.005), tumor size(HR = 6.657, 95% CI:2.343–18.910, *P* < 0.001), TN(HR = 2.967, 95% CI:1.401–6.282, *P* < 0.001), elderly patients(HR = 3.212, 95% CI:1.684–4.698, *P* = 0.002), and WHO/ISUP grade(HR = 4.035, 95% CI:1.075–15.137, *P* = 0.039) were independent prognostic factors for CSS(Table 4).

**Table 2:**
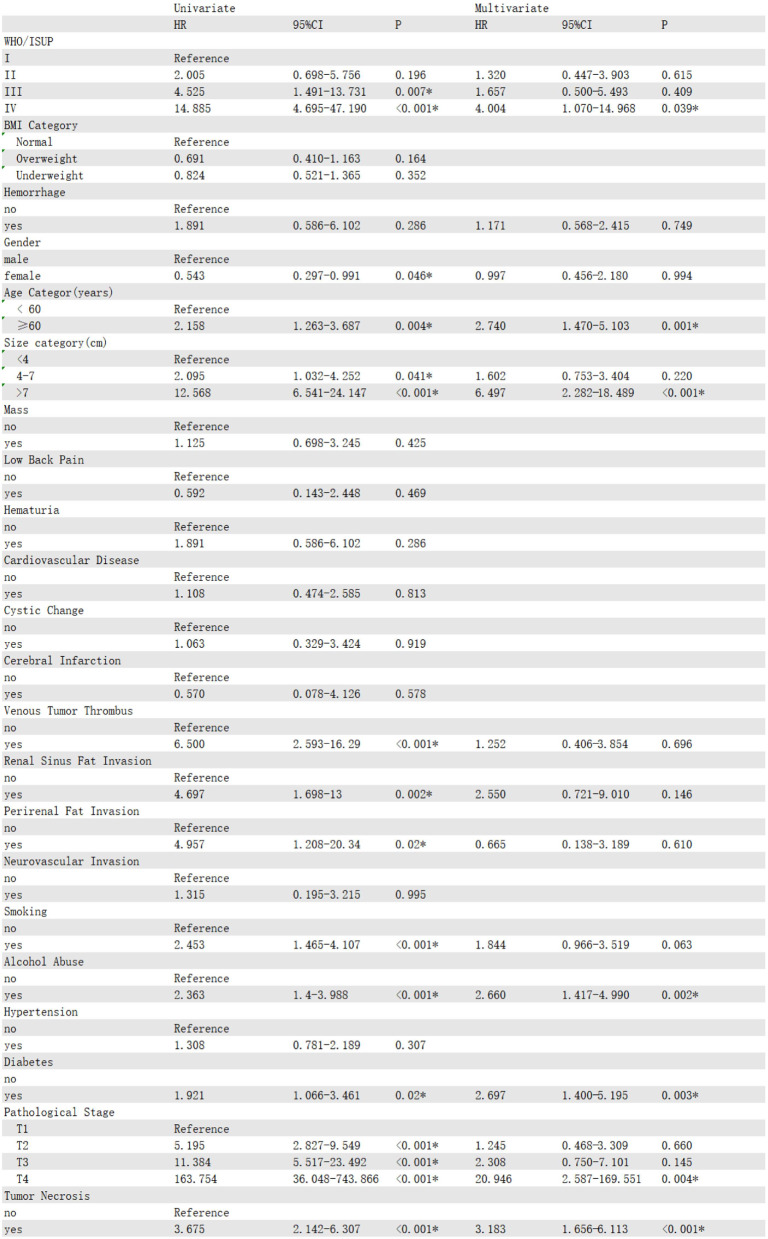
Univariable and multivariable Cox regression analysis of OS for ccRCC patients in the training cohort

**Table 3:**
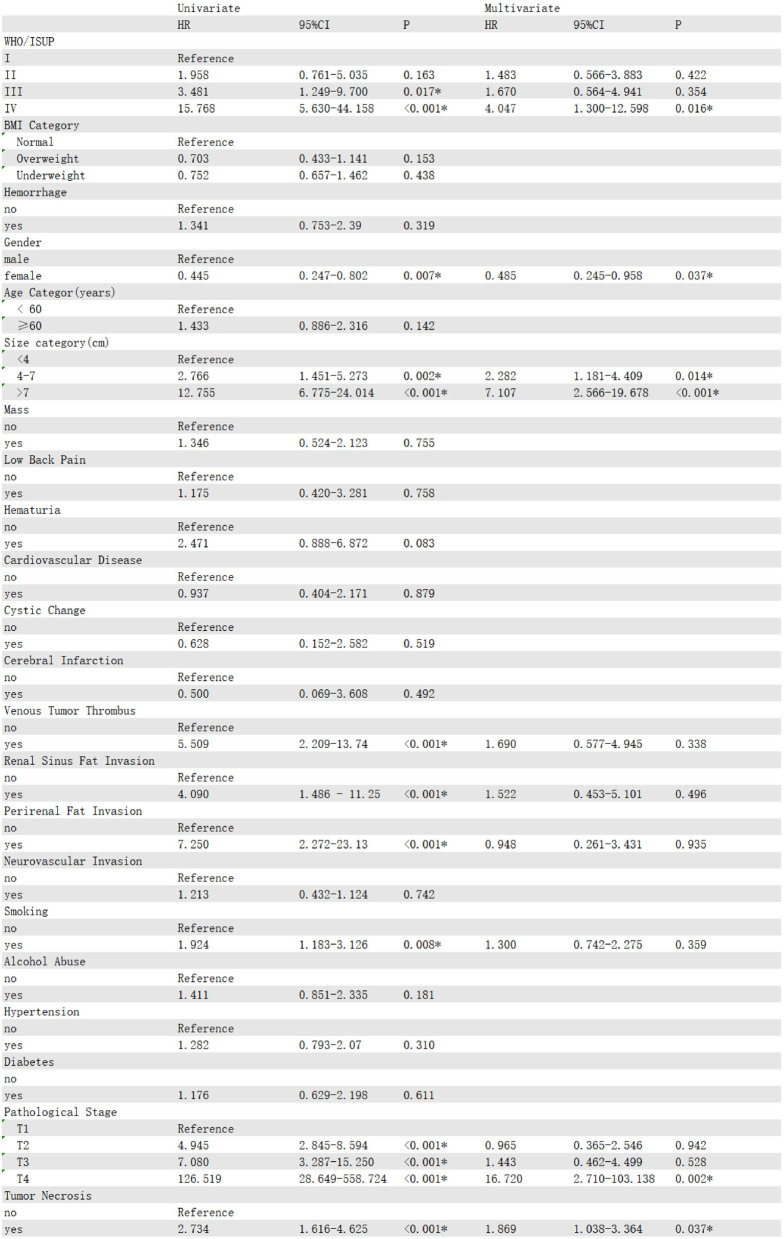
Univariable and multivariable Cox regression analysis of RFS for ccRCC patients in the training cohort

**Table 4:**
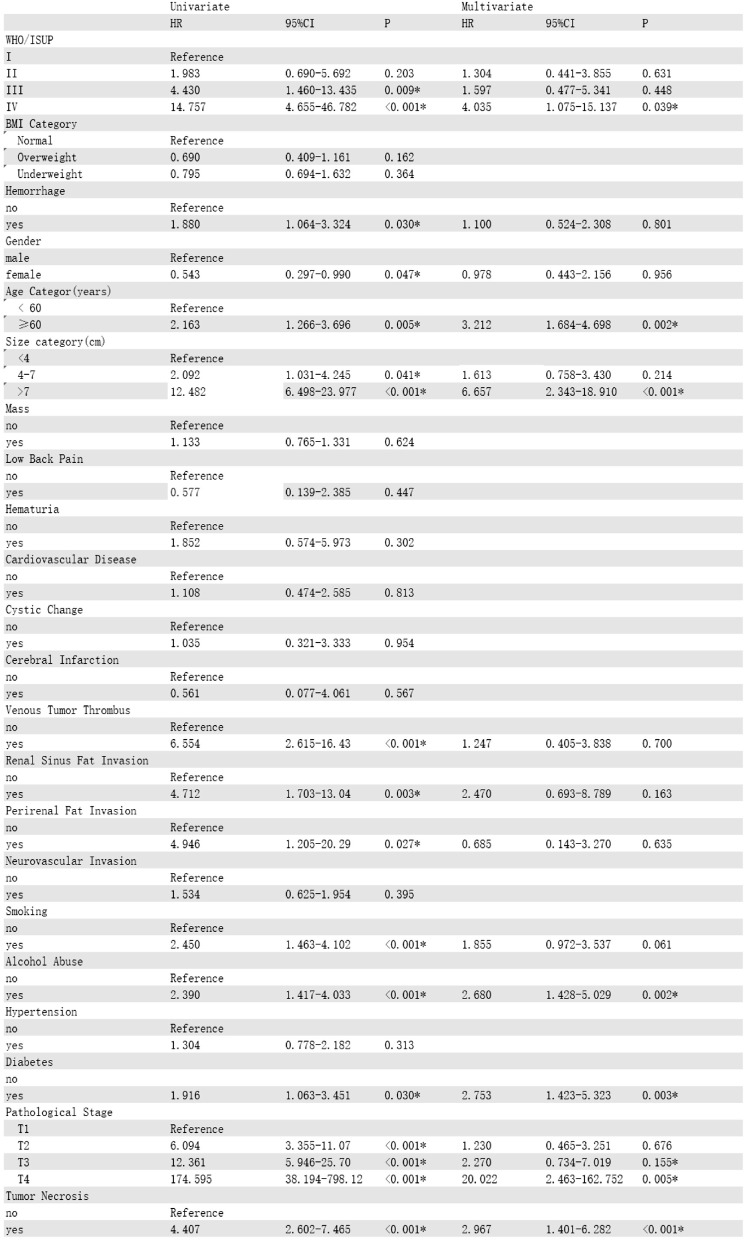
Univariable and multivariable Cox regression analysis of CSS for ccRCC patients in the training cohort

### Construction of nomograms for predicting 1 - year, 3 - year, and 5 - year OS, RFS, and CSS

**Fig. 2:**
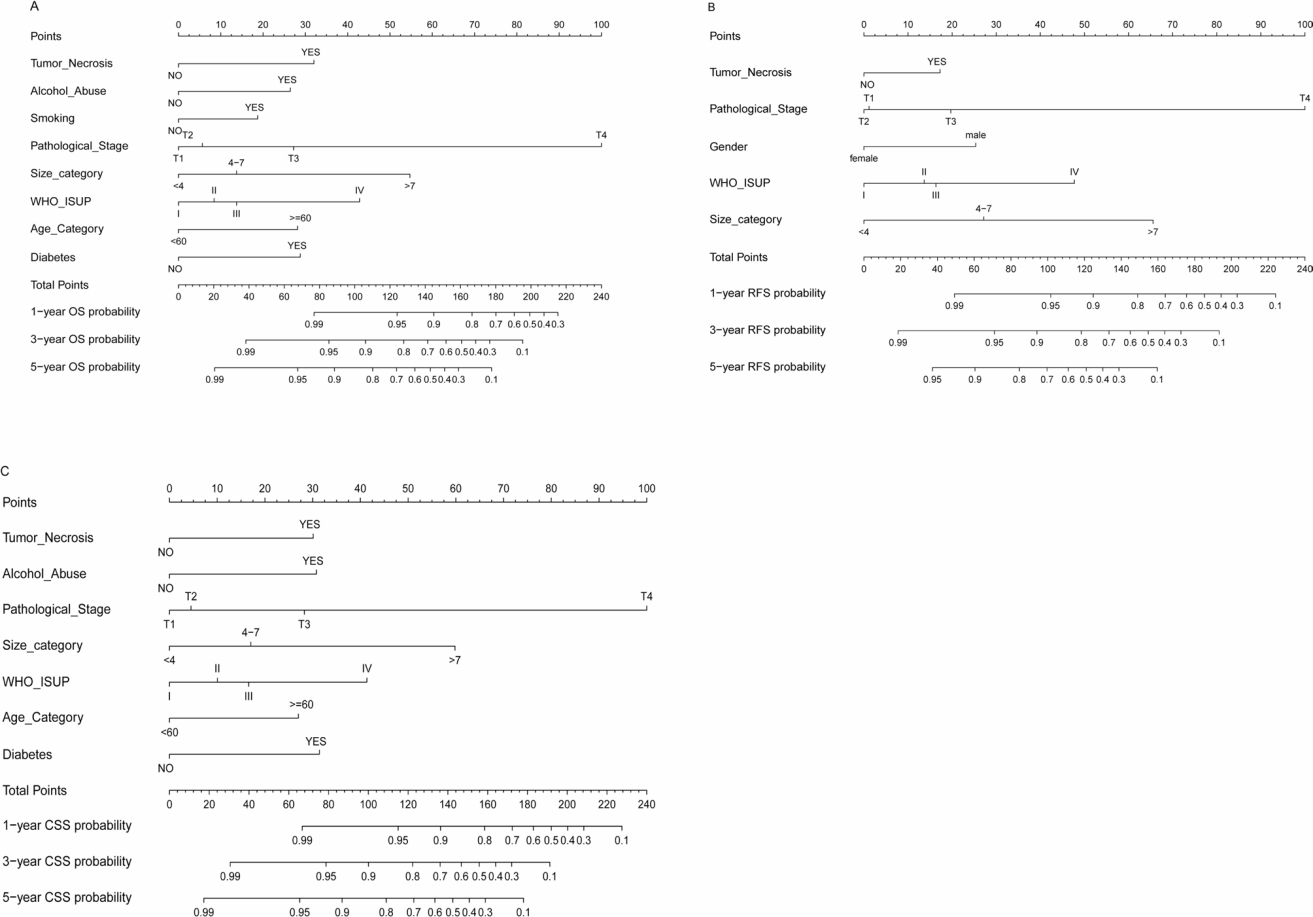
Nomograms for the prognosis in patients with ccRCC (Age, years; Tumor size, cm). **A** Nomogram for prediction of 1-, 3-, and 5-year OS. **B** Nomogram for prediction of 1-, 3-, and 5-year RFS(**C**) Nomogram for prediction of 1-, 3-, and 5-year CSS

**Fig. 3:**
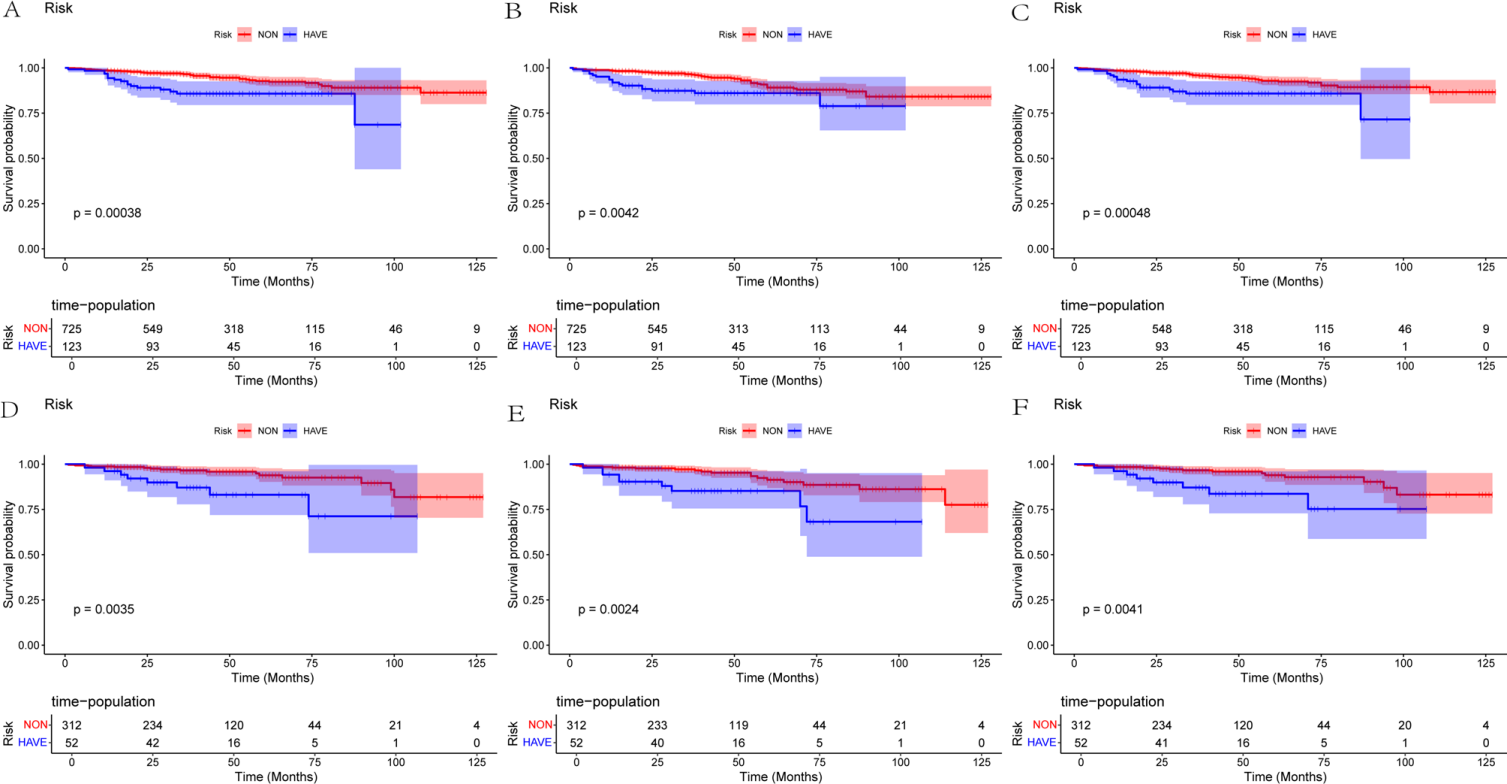
Kaplan-Meier curves of OS, RFS and CSS for patients in the non- and have Tumor necrosis. **A** Kaplan-Meier curves of OS for patients in the non- and have Tumor necrosis in the training cohort. **B** Kaplan-Meier curves of RFS for patients in the non- and have Tumor necrosis in the training cohort. **C** Kaplan-Meier curves of CSS for patients in the non- and have Tumor necrosis in the training cohort. **D** Kaplan-Meier curves of OS for patients in the non- and have Tumor necrosis in the validation cohort. **E** Kaplan-Meier curves of RFS for patients in the non- and have Tumor necrosis in the validation cohort. **F** Kaplan-Meier curves of CSS for patients in the non- and have Tumor necrosis in the validation cohort

Nomograms were constructed using the independent prognostic factors selected by multivariate Cox analysis and the optimal model variables chosen by stepwise Cox regression, and the corresponding scores for each parameter were listed. The nomograms could predict the 1 - year, 3 - year, and 5 - year OS. In the nomogram, the length of the line for each variable represented its contribution to OS. The longer the line, the greater the contribution. As shown in the nomogram, T stage was the strongest prognostic factor for OS, followed by tumor size and WHO/ISUP grade. Meanwhile, TN was also an important variable for the OS of patients, which was significantly higher than smoking, Alcohol abuse, diabetes, and elderly patients (Fig. 2A). Additionally, we constructed a nomogram to predict the CSS of patients, and the result was similar to the OS nomogram (Fig. 2C). It is noteworthy that the result of the RFS nomogram showed that the line length of TN was the shortest, which was different from the OS and CSS nomograms (Fig. 2B).To clarify TN prognostic impact, we generated Kaplan - Meier (KM) curves to visualize survival differences between patients with (HAVE, blue) and without (NON, red) TN across OS, (Figs. 3A, D), PFS, (Figs. 3B, E), and CSS (Figs. 3C, F) in training and validation cohorts. For statistical comparison, we applied the log - rank test for comparing survival distributions between groups.For OS, PFS, and CSS, KM curves showed clear separation: TN - positive groups had steeper survival probability declines. Log - rank tests confirmed significant differences (OS: training *P* = 0.00038, validation *P* = 0.0035; PFS: training *P* = 0.00042, validation *P* = 0.0024; CSS: training *P* = 0.0048, validation *P* = 0.0041). TN consistently acted as an adverse prognostic factor, with TN -positive patients facing higher death, progression, and cancer - specific mortality risks.

### Nomogram validation

In the training cohort and the validation cohort, the C - indices of the OS nomogram were 0.885 and 0.855, respectively. Since C-index values range from 0 to 1, with values > 0.7 indicating good discriminative ability in survival models, these results clearly indicate that the OS nomogram had excellent discriminability. The C - indices of the RFS nomogram in training cohort and validation cohort were 0.821 and 0.724, and those of the CSS nomogram were 0.877 and 0.870, all exceeding the 0.7 threshold, confirming robust discriminative power across all survival endpoints.

**Fig. 4:**
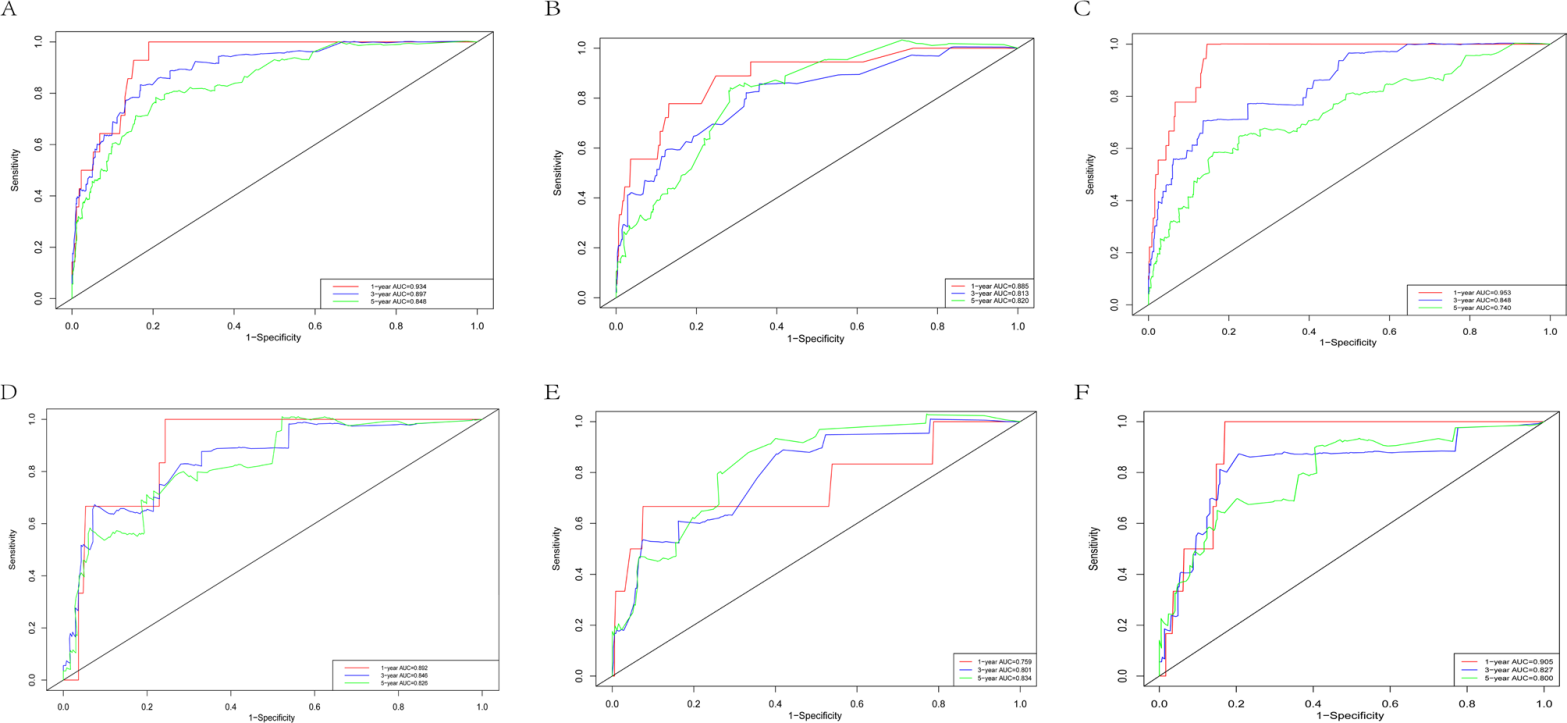
The ROC for 1-, 3- and 5-year prognosis. **A** The ROC of nomogram for OS in training cohort. **B** The ROC of nomogram for OS in validation cohort. **C** The ROC of nomogram for RFS in training cohort. **D** The ROC of nomogram for RFS in the validation cohort. **E** The ROC of nomogram for CSS training cohort. **F** The ROC of nomogram for CSS validation cohort

In the training cohort, the time-dependent AUC values of the OS nomogram for 1-year, 3-year, and 5-year survival were 0.934, 0.897, and 0.848 (Fig. 4A), while in the validation cohort, they were 0.885, 0.813, and 0.820 (Fig. 4B). AUC values > 0.8 are generally considered to reflect high predictive precision, and these results—consistently above 0.8 across time points and cohorts—demonstrate strong short- and long-term predictive performance for OS. For the RFS nomogram, the AUC values were 0.963, 0.848, and 0.740 in the training cohort (Fig. 4C), and 0.882, 0.845, and 0.825in the validation cohort (Fig. 4D).While the training cohort’s 5-year RFS AUC dipped slightly, the validation cohort maintained 3- and 5-year values above 0.8, highlighting stable performance in stratifying short-term recurrence risk (excellent at 1 year) and reliable mid- to long-term recurrence prediction—key for tailoring surveillance intensity or adjuvant therapy. For CSS, the AUC values were 0.759, 0.801, 0.834 in the training cohort and 0.905, 0.827, 0.800 in the validation cohort (Fig. 4E, F). In the training cohort, we observed a gradual improvement in AUC over follow-up duration, likely due to diminished competing risks of non-cancer-related deaths in longer-term follow-up, which enhances the model’s ability to isolate cancer-specific mortality risk.Collectively, these results demonstrated that the nomograms had high predictive ability across all survival endpoints.

**Fig. 5:**
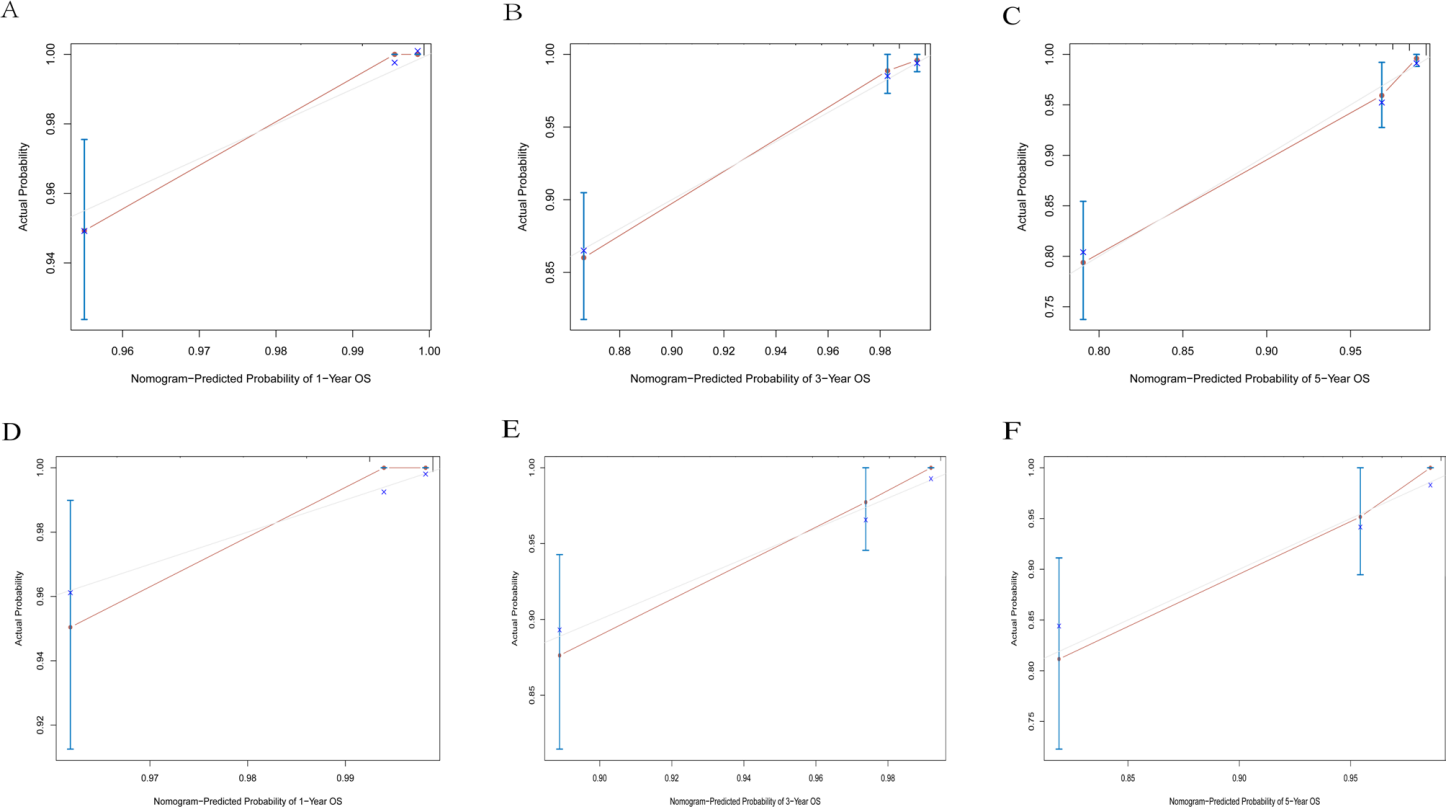
Calibration curves of the nomogram. **A** 1-year OS in the training cohort. **B** 3-year OS in the training cohort. **C** 5-year OS in the training cohort. **D** 1-year OS in the validation cohort. **E** 3-year OS in the validation cohort. **F** 5-year OS in the validation cohort

**Fig. 6:**
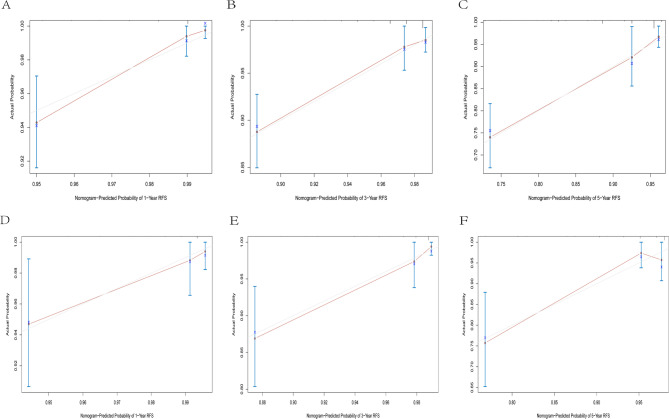
Calibration curves of the nomogram. **A** 1-year RFS in the training cohort. **B** 3-year RFS in the training cohort. **C** 5-yearRFS in the training cohort. **D** 1-year RFS in the validation cohort. **E** 3-year RFS in the validation cohort. **F** 5-year RFS in the validation cohort

**Fig. 7:**
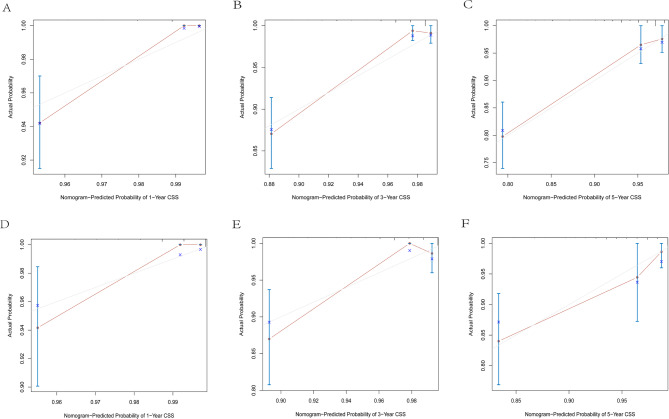
Calibration curves of the nomogram. **A** 1-year CSS in the training cohort. **B** 3-year CSS in the training cohort. **C** 5-year CSS in the training cohort. **D** 1-year CSS in the validation cohort. **E** 3-year CSS in the validation cohort. **F** 5-year CSS in the validation cohort

To further evaluate prediction accuracy, calibration curves at 1 - year, 3 - year, and 5 - year were used to compare the predicted survival probabilities with the actually observed ones. The results showed that the predicted curves closely overlapped with the diagonal lines in both the training cohort and the validation cohort, with minimal deviations between predicted and observed values. This strong alignment indicates that the nomograms had high prediction accuracy for OS, CSS, and RFS (Figs. 5, 6 and 7),complementing the discriminative performance shown by AUC and validating that the models not only distinguish risk groups but also provide trustworthy estimates to guide clinical decisions.

### Clinical application of the nomogram

**Fig. 8:**
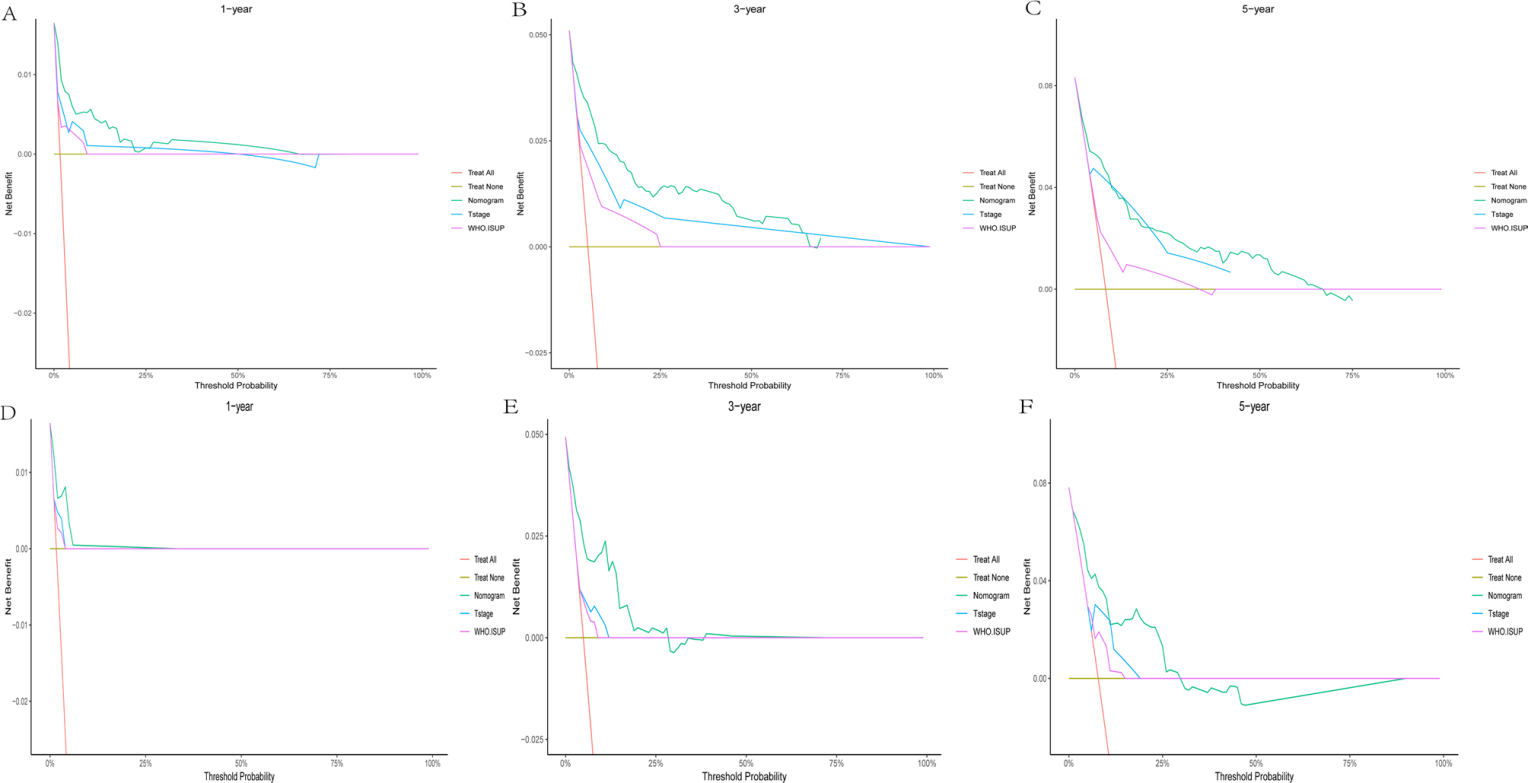
Decision curves of the nomogram predicting OS in the training and validation cohort. **A** DCA curves of the nomogram predicting1-year OS in the training cohort. **B** DCA curves of the nomogram predicting 3-year OS in training cohort. **C** DCA curves of the nomogram predicting 5-year OS in the training cohort. **D** DCA curves of the nomogram predicting 1-year OS in validation cohort. **E** DCA curves of the nomogram predicting 3-year OS in validation cohort. **F** DCA curves of the nomogram predicting 5-year OS in validation cohort

**Fig. 9:**
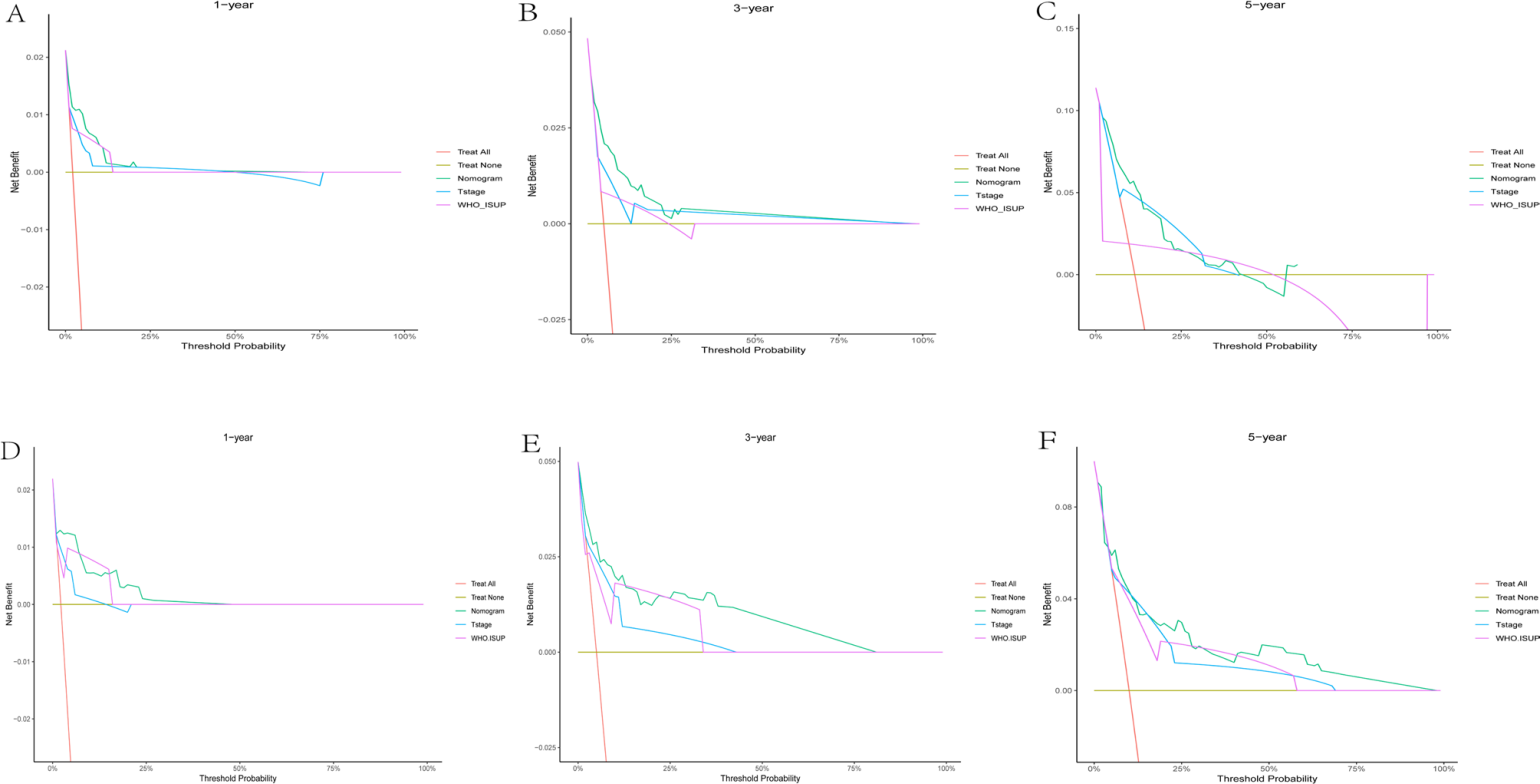
Decision curves of the nomogram predicting RFS in the training and validation cohort. **A** DCA curves of the nomogram predicting1-year RFS in the training cohort. **B** DCA curves of the nomogram predicting 3-year RFS in training cohort. **C** DCA curves of the nomogram predicting 5-year RFS in the training cohort. **D** DCA curves of the nomogram predicting 1-year RFS in validation cohort. **E** DCA curves of the nomogram predicting 3-year RFS in validation cohort. **F** DCA curves of the nomogram predicting 5-year RFS in validation cohort

**Fig. 10:**
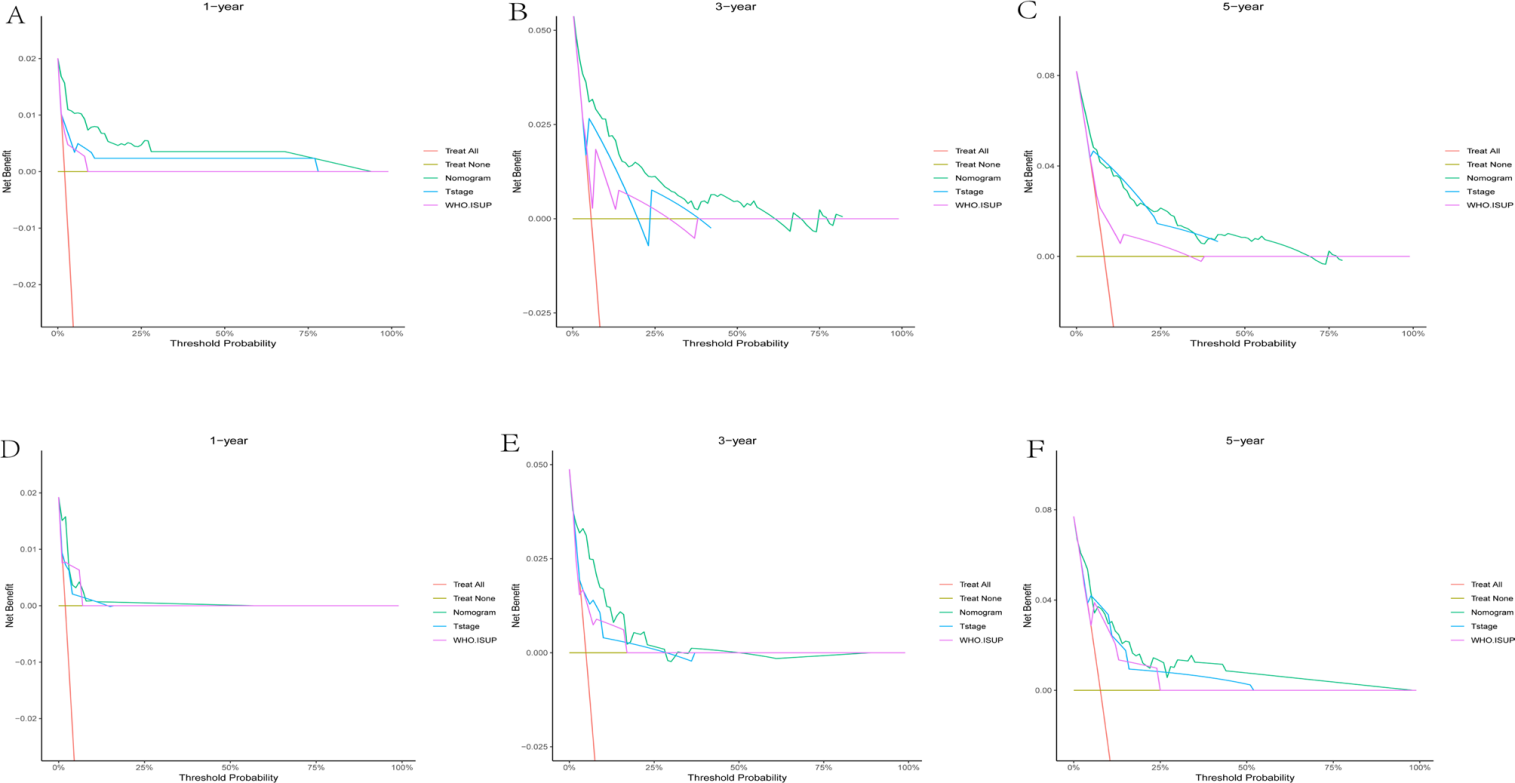
Decision curves of the nomogram predicting CSS in the training and validation cohort. **A** DCA curves of the nomogram predicting1-year CSS in the training cohort. **B** DCA curves of the nomogram predicting 3-year CSS in training cohort. **C** DCA curves of the nomogram predicting 5-year CSS in the training cohort. **D** DCA curves of the nomogram predicting 1-year CSS in validation cohort. **E** DCA curves of the nomogram predicting 3-year CSS in validation cohort. **F** DCA curves of the nomogram predicting 5-year CSS in validation cohort

**Fig. 11:**
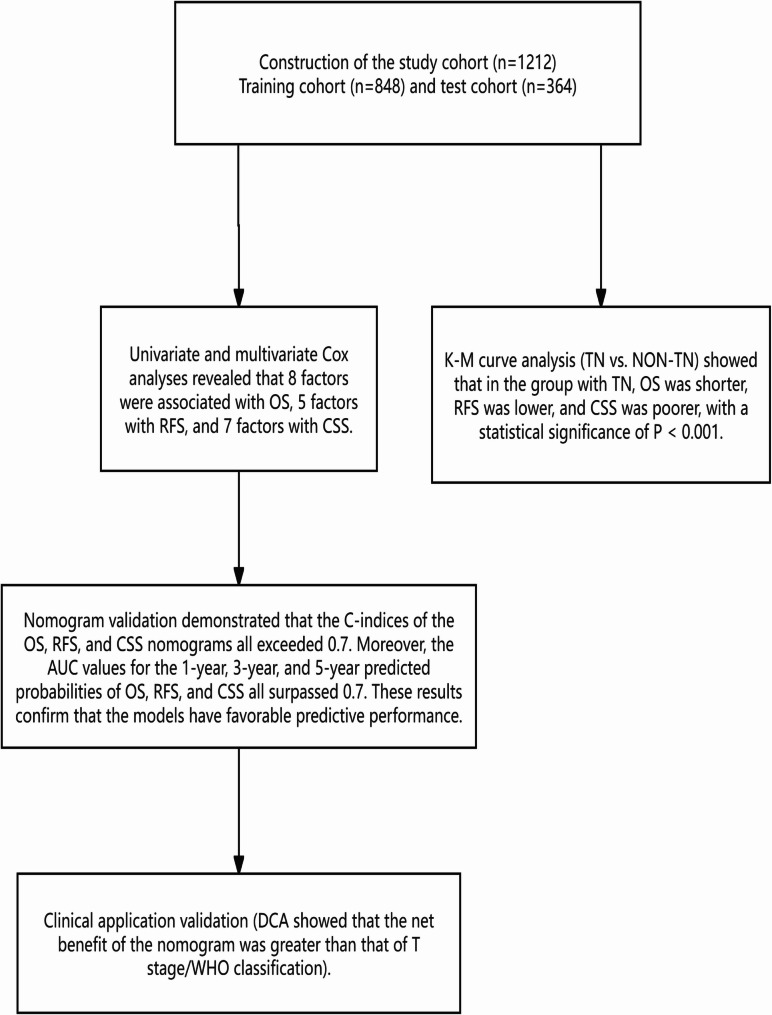
The flowchart of analytical process

To evaluate the clinical utility of our nomogram, we performed DCA by integrating tumor T stage and WHO/ISUP grade (key prognostic factors with the highest weights in the nomogram, as indicated by their longest line lengths). Across 1 - year, 3 - year, and 5 - year time horizons (Figs. 8, 9 and 10), the DCA results consistently showed that for ccRCC patients, using the nomogram to guide clinical decisions yielded greater net benefits for OS, CSS, and RFS compared to relying solely on T stage or WHO/ISUP grade.For example, in the 1 - year OS analysis (Figs. 8A, 9A and 10A), the nomogram net benefit curve outperformed the curves for T stage and WHO/ISUP grade across most threshold probabilities.Similar trends were observed for 3 - year (Figs. 8B, 9B and 10B) and 5 - year (Figs. 8C, 9C and 10C) OS, as well as for CSS and RFS across all three cohorts.The validation cohort (panels D-F of 8–10 figure) further confirmed the nomogram clinical value. The DCA curves demonstrated that the nomogram maintained superior net benefits over T stage and WHO/ISUP grade in an independent patient population, highlighting its generalizability. Collectively, these findings indicate that the nomogram can assist clinicians in making more precise risk - based decisions, such as selecting appropriate candidates for adjuvant therapy or determining optimal surveillance frequencies, ultimately enhancing personalized care for ccRCC patients.Finally, we drew an analysis result flowchart to visually summarize the model framework and the key steps of model verification.(Fig. 11).

## Discussion

Currently, the impact of TN on the prognosis of renal cell carcinoma remains controversial [15, 27]. Some believe that TN is related to prognosis, while other studies suggest that TN is a negative predictor of prognosis. In this study, we incorporated numerous clinicopathological factors, such as whether there was renal sinus and perirenal fat invasion reported in the postoperative pathology, and whether there was venous tumor thrombus. The results demonstrated that the proposed nomogram exhibited high predictive performance in the validation cohort.This retrospective study analyzed clinicopathological data from 1,212 patients with pathologically confirmed ccRCC over a 10-year period (2013–2023) to evaluate the prognostic significance of TN.

This study showed that TN was of significant importance for the OS, CSS, and RFS of ccRCC patients. TN demonstrated significant prognostic independence, retaining predictive value beyond established clinicopathological parameters such as tumor diameter, Fuhrman nuclear grade, and TNM stage in multivariable analysis. Notably, we found that TN, T stage, WHO/ISUP grade, and tumor size were key prognostic factors. Diabetes, alcohol abuse, age, and gender also influenced the prognosis and disease progression of patients. In this study, we constructed three nomograms to predict the prognosis of ccRCC patients. The combined application of these indicators could provide a more comprehensive basis for individualized prognostic assessment. In clinical research, clinicians and researchers frequently utilize TNM staging and WHO/ISUP grade to evaluate patients’ prognosis and make clinical decisions. However, recent studies have shown that compared with the traditional TNM staging system, nomograms constructed based on clinicopathological data have higher accuracy in predicting patient survival [35]. Therefore, urologists can utilize these nomograms to assess the prognosis of ccRCC patients, formulate effective and individualized treatment strategies, and thus reduce the risk of death.

For localized clear cell renal cell carcinoma, the tumor stage and lymph node metastasis status in the TNM staging system are important prognostic factors. The tumor stage takes into account the size of the tumor and the extent of the disease. Lymph node metastasis is relatively rare in renal cell carcinoma, but it is always associated with a poor prognosis [36]. In addition to TNM staging, a higher WHO/ISUP grade of the tumor is also a risk factor for poor prognosis [9]. Currently, many risk factors have been identified, such as tumor grade, histological features, performance status, clinical symptoms, margin status, etc [37].

Diabetes or hyperglycemia has been proven to promote the proliferation and metastasis of tumor cells. An increase in the level of insulin - like growth factor (IGF) − 1 can induce cell proliferation and inhibit apoptosis simultaneously [38, 39], which is consistent with the results of our study.

The poor prognosis of elderly patients may be attributed to cardiovascular diseases caused by the loss of renal function after surgery [40], as well as immune senescence and immunosuppression related to the immune system after surgery [41].

Tumor size also impacts the overall prognosis of patients, which is a research hotspot. Some studies have indicated that ccRCC patients with a larger tumor diameter tend to have a worse prognosis compared to those with a smaller one [42], it corresponds to the outcomes of our research. A larger tumor diameter represents stronger invasiveness [43]. It has been found that TN is closely related to tumor size. A reasonable explanation for this is that due to the rapid growth of the tumor, the microvasculature experiences ischemia and damage as a result of overloaded supply, leading to TN. In breast cancer, TN is associated with an increase in tumor size [24]. In experimental mouse models [44], an increase in mass is related to hypoxia.

Notably, gender factors play a relatively important role in the RFS of ccRCC. Hiroshi Fukushima et al. retrospectively reviewed 2055 patients with cT1–4N0M0 ccRCC who underwent partial or radical nephrectomy in the International Renal Cancer Markers Consortium (INMARC) dataset. They found that the RFS of females was significantly better than that of males [45]. Some research has revealed that female patients with renal cell carcinoma under 58 years old have a survival advantage over males, while this survival advantage disappears in female patients aged 59 and above [46]. This may indicate that sex hormones have a certain influence on the occurrence and development of renal cell carcinoma.This is consistent with the results of our study, and being female is a favorable factor for the RFS of ccRCC.

Currently, the mechanism of the relationship between TN and the prognosis of solid tumors remains unclear. There is a hypothesis that the rapid proliferation of cells beyond the vascular system leads to tumor hypoxia, resulting in subsequent tumor cell death and promoting the metastatic cascade [47, 48]. Necrosis serves as a histological manifestation of hypoxic microenvironments within tumors, representing a pathophysiological consequence common across human cancers [49]. Hypoxic microenvironments promote genomic instability and disrupt DNA repair mechanisms, rendering these tumor regions therapeutically recalcitrant. Clinically, hypoxia correlates with poorer solid tumor outcomes, enhanced dissemination capacity, and resistance to chemo-radiotherapy [50–52]. Furthermore, hypoxia-inducible factor-1α (HIF-1α) mediates transcriptional reprogramming that drives invasive phenotypes [53].Furthermore, some studies suggest that necrosis is an uncontrolled form of cell death, where the cell membrane ruptures, releasing the contents of the cell and triggering an inflammatory response [54, 55]. TN attenuates local inflammatory infiltration while triggering systemic inflammation [56]. Key mechanisms include cyclooxygenase product accumulation [57] and nitric oxide generation at lesion sites, driving concurrent proliferation and cell death. These events associate with epigenetic silencing via promoter hypermethylation in critical tumor-suppressive and apoptotic genes [58, 59]. Resultant apoptotic impairment shifts cell death toward necrosis, releasing oncogenic cellular components that fuel cancer advancement [60]. In addition, necrosis may be a marker of activated proliferation, and subsequent anti - apoptotic pathways in tumors are markers of cancer progression [61].

A meta - analysis, which included a total of 14,084 renal cell carcinoma patients, showed that histopathological TN was associated with poorer CSS, OS, and PFS in RCC patients, demonstrating that TN is an important prognostic factor for renal cell carcinoma [28]. Similarly, a study using the Korean Renal Cancer Study Group (KRoCS) Database to investigate the one - year recurrence rate after renal cell carcinoma surgery confirmed that TN was an independent predictor of renal cell carcinoma recurrence, this aligns with the findings of our study [62].

Currently, adjuvant immunotherapy has reshaped the management of high-risk localized RCC. The KEYNOTE-564 trial by Choueiri et al. demonstrated that pembrolizumab reduces the risk of recurrence by 32% [63], with long-term follow-up showing an 8.2% improvement in 5-year overall survival [64]; however, its precise application depends on risk stratification. Grunwald et al.’s Delphi study noted that the current use of Leibovich scores for patient selection still requires optimization, with a need to integrate additional biomarkers [65]. In our study, TN was identified as an independent prognostic factor in ccRCC, and the constructed nomogram showed high concordance with the “high-risk patients” defined in KEYNOTE-564. Notably, the high-risk TN subgroup corresponded exactly to the population with the most significant benefits from pembrolizumab [63, 64], suggesting that TN may complement existing scoring systems to identify high-risk patients underestimated by traditional criteria [65].​.

Throughout the treatment course, Grunwald et al. confirmed that the modified Glasgow Prognostic Score (mGPS) at progression can screen for patients who derive sustained benefits from immunotherapy [66], while our study found a significant association between TN and expert-defined “refractory recurrence within 6 months of immune checkpoint inhibitor (ICI) therapy” [65]. Since TN and mGPS reflect local invasiveness and systemic inflammatory status respectively, their combined application may further optimize clinical decision-making [65].Our clinically applicable nomogram integrates readily available demographic and clinicopathologic variables. In both the training cohort and the validation cohort, the nomogram showed high C-index and AUC values, indicating its high accuracy. The calibration curve also demonstrated excellent predictive performance. In terms of clinical applicability, our DCA confirmed that the clinical net benefit of the nomogram exceeded that of the tumor T stage and the WHO/ISUP grading system.

To the best of our knowledge, this study is the first to construct a nomogram integrating TN for prognostic analysis in ccRCC, which represents a notable strength. Several key merits of this research should be emphasized: Firstly, we systematically explored the prognostic value of TN through multi-dimensional analyses, including Kaplan-Meier curves, univariate and multivariate Cox regression, confirming its independent association with OS, RFS, and CSS, which provides robust evidence for TN as a critical prognostic biomarker. Secondly, the constructed nomograms demonstrated excellent predictive performance, with C-indices and time-dependent AUC values all exceeding 0.7 in both training and validation cohorts, indicating strong discriminative ability. Thirdly, rigorous validation strategies were employed, including internal validation with calibration curves showing close alignment between predicted and observed outcomes, and DCA confirming higher net clinical benefit compared to traditional indicators like T stage and WHO classification, supporting the practical utility of the models.​.

However, this study also has several limitations that should be acknowledged. Firstly, as a retrospective study, inherent selection bias and confounding bias are inevitable. Secondly, this is a single-center study with a medium-sized sample size, which may limit the generalizability of our findings to broader patient populations. Further large-scale, multi-center prospective studies are therefore needed to validate the clinical significance of TN in ccRCC prognosis. Thirdly, regarding data processing, we addressed lost-to-follow-up cases by excluding them from the final analysis cohort, as incomplete outcome datacould compromise the stability of prognostic models. For missing values in baseline clinical features or biomarkers, a complete case analysis strategy was adopted, including only patients with no missing key variables for model development and validation. While this approach simplifies data analysis and is commonly used in retrospective studies, it may reduce sample size and introduce selection bias if missing data are not completely random. To mitigate this risk, we evaluated missing patterns before exclusion, finding low missing rates for key variables (< 5%) with no significant differences between training and validation cohorts, suggesting controllable bias.Furthermore, although we validated the prognostic nomogram using an internal validation cohort—with the model demonstrating favorable discriminative ability and calibration in both training and validation sets—potential overfitting risks should still be noted. Specifically, the internal validation cohort and training set were derived from the same center, and their homogeneity in patient baseline characteristics and clinical data collection standards may have caused the model to overfit to specific population features, thereby impairing its generalizability to externally heterogeneous populations. Additionally, despite strict definition of quantification criteria for biomarkers such as TN, potential biases in retrospective data may have been learned and amplified by the model, which could compromise prediction stability. Moreover, while the sample size meets basic requirements for model development, the limited number of events may have led the model to over-capture extreme values or spurious associations. Future studies will therefore focus on multi-center external validation to confirm the model’s generalizability and expand the sample size to enhance its capacity to capture real-world clinical patterns.

## Conclusion

In conclusion, the TN reported in postoperative pathology can serve as an independent prognostic indicator for the OS, CSS, and RFS of non - metastatic ccRCC patients after surgery. Based on this, a new nomogram for predicting ccRCC patients was constructed. The nomogram achieved superior discrimination performance, facilitating evidence-based risk assessment for personalized treatment planning and follow-up scheduling. Prospective validation in diverse cohorts remains essential to confirm clinical utility.

## Data Availability

The data that support the findings of the study are available from the corresponding author upon reasonable request.

## Abbreviations

RCC: Renal cell carcinoma
ccRCC: Clear cell renal cell carcinoma
TN: Tumor necrosis
ROC: Receiver operating characteristic
HR: Hazard ratio
CI: Confidence interval
OS: Overall survival
CSS: Cancer-specific survival
AUC: Area under the ROC curve
C-index: Concordance index
DCA: Decision curve analysis
